# The use of optimised heating trousers and the role of the blood flow on the reduction in muscle temperature post warm up

**DOI:** 10.1186/2046-7648-4-S1-A77

**Published:** 2015-09-14

**Authors:** Margherita Raccuglia, Alex Lloyd, Davide Filingeri, Simon Hodder, George Havenith

**Affiliations:** 1 Environmental Ergonomics Research Centre, Loughborough University, Loughborough UK

## Introduction

Activities that are highly dependent on power output can benefit from increases in muscle temperature (T_m_) in terms of work done and skeletal muscle power output. When athletes experience a significant delay between active warm up and performance, T_m _declines. Previous studies have demonstrated that using heated trousers during a period of inactivity can attenuate this decline, with a greater peak power output as result [1,2]. However, in these studies, the reduction in T_m _was not completely eliminated. Thus, in the current study we aimed to optimise the heating procedure, in order to eliminate the reduction in T_m _post-warm up. Furthermore, to understand the reason of this reduction, the effect of the blood flow in the cooling process of the leg was studied.

## Method

Ten male cyclists participated in this experiment. The heating garment was applied during 30 minutes of passive recovery following 15 minutes of active warm up. The heating procedure was optimised by using water perfused trousers with an adjusted water temperature of 43 °C. The effect of the blood flow was observed during the recovery period using full restriction of arterial and venous blood flow in one leg (OCCLUDED), while the other leg was used as control (CONTROL). T_m _of the *vastus lateralis *was measured at three different depths beyond the muscle fascia: 5 mm, 15 mm and 25 mm.

## Results

During the passive recovery, blood flow significantly reduced (p < 0.05) T_m _in CONTROL compared to OCCLUDED condition by 0.33(0.3) °C. In the CONTROL condition the heating procedure significantly increased T_m _(p < 0.05) by 1(1.3) °C at 5 mm depth and there was only a small reduction (p < 0.05) in T_m _of 0.1(0.8) °C and 0.1(0.3) °C at 15 mm and 25 mm, respectively. The use of the optimised heating trousers coupled with the removal of the blood flow resulted in a T_m _increase (p < 0.05) of 1.8(1.6) °C, 0.6(0.7) °C and 0.2(0.3) °C at 5 mm, 15 mm and 25 mm depth, respectively. Compared to the previous muscle warming method, the current approach resulted in a 0.61 °C warmer muscle (15 mm depth) at the end of the recovery period.

## Conclusion

By optimising the heating procedure, using water perfused trousers with temperature of 43 °C, it is possible to maintain T_m _during period of inactivity following an active warm up. Blood flow was identified as a contributor to the earlier observed reduction in T_m _post warm up.
